# The Impact of a Novel Methodological Process for Needling Scars, Fascia, and Muscles in the Management of Myofascial Dysfunction and Chronic Pain in a Population Living With Social and Health Inequities: Quantitative Findings From a Longitudinal Observational Pilot Study

**DOI:** 10.1155/prm/8567447

**Published:** 2025-09-28

**Authors:** Ly Nguyen, Barb L. Eddy, Nicholas West, Jeffrey N. Bone, Leanne M. Currie, Gillian Lauder

**Affiliations:** ^1^Department of Family and Community Practice, Vancouver Coastal Health, Vancouver, British Columbia, Canada; ^2^School of Nursing, The University of British Columbia, Vancouver, British Columbia, Canada; ^3^Department of Nurse Practitioners, Vancouver Coastal Health, Vancouver, British Columbia, Canada; ^4^Research Institute, BC Children's Hospital, Vancouver, British Columbia, Canada; ^5^Department of Anesthesiology, Pharmacology & Therapeutics, The University of British Columbia, Vancouver, British Columbia, Canada

**Keywords:** chronic pain, myoActivation, myofascial dysfunction, needling, nonpharmacological, primary care, social and health inequities

## Abstract

**Background:** Nonpharmacological interventions are necessary tools for managing chronic pain to reduce dependence on prescribed analgesic medications. myoActivation® is an innovative systematic nonpharmacological assessment and needling process to help reduce chronic pain related to myofascial dysfunction (MFD).

**Aim:** Analyze quantitative data collected during a longitudinal mixed methods observational pilot study in patients living with social and health inequities undergoing myoActivation as part of routine clinical care to determine the impact of this treatment approach on pain intensity, enjoyment of life, general activity, and unregulated drug use.

**Methods:** Following ethics approval, we conducted a prospective observational study of patients receiving sequential myoActivation for chronic pain. Data were collected at baseline (Week 1) and subsequently at 4, 12, and 24 weeks using validated tools, including the PEG (Pain, Enjoyment of Life, General Activity) scale as well as self-reports of substance and analgesic use.

**Results:** There were 35 participants with a median (interquartile range) baseline PEG score of 7.7/10 (6.7–8.7/10). PEG scores improved at each follow-up, with a mean difference from baseline of −2.5 (95% CI −3.4 to −1.5, *p* < 0.001) at Week 24, which corresponds to a clinically significant (> 30%) and lasting improvement. At 24 weeks, 9/27 (33%) participants reported less unregulated drug use, and 8/27 (30%) reported less analgesic medication use.

**Discussion:** This study suggests that myoActivation pain care can be an effective tool, with a lasting positive impact, to manage MFD and chronic pain in a population living with social and health inequities. Further studies are needed to examine the impact of myoActivation in the primary care setting.

**Trial Registration:** ClinicalTrials.gov identifier: NCT04261959

## 1. Introduction

Myofascial dysfunction (MFD) and chronic pain occur in response to past injuries, surgeries, or repetitive strain injuries; poor posture; nutritional deficiencies; lack of sleep; mental health issues; inflammatory conditions; and other coexisting diseases. These factors all play a role in the development of stiffness in the myofascial soft tissues resulting from fascial tension and muscles in sustained contraction. MFD is misunderstood and commonly overlooked as a contributing cause of chronic pain [1–3]. There is currently little consensus on definitions and criteria for MFD versus myofascial pain syndrome in the published literature [4]. For the purposes of this work, MFD was determined by identifying significant scars, skin under tension, palpable painful fascial densities, palpable painful muscle trigger points, or a combination of these factors [5–10]. This is not the same as the widely discussed myofascial pain syndrome [11].

myoActivation® is an innovative, systematic process designed to assess and resolve the myofascial components of chronic pain. The assessment components include a history of lifetime trauma, posture assessment, observation of the patient performing a standardized set of movement tests, skin inspection, and palpation. The therapeutic intervention is a nonpharmacological needling technique utilizing fine-gauge cutting tip needles to release scars and myofascial tension [5, 6, 12, 13]. An outline of the myoActivation approach is presented in the Methods Section, “Clinical procedures: myoActivation® assessment and treatment,” with further details in the supporting document (available here), The myoActivation Process.

People living with social and health inequities have increased risk of chronic pain and are adversely affected by limited access to appropriate pain care [14–17]. The Downtown East Side (DTES) in Vancouver, British Columbia, Canada, is home to one of the most complex populations living with social and health inequities. People in the DTES are likely to have experienced and continue to experience violence, trauma, poverty, food insecurity, poor housing, homelessness, poor sleep, mental health disorders (e.g., anxiety, depression, posttraumatic stress disorder [PTSD], psychosis), medical trauma [18–22], chronic disease, and chronic pain.

Unregulated drug use, substance use disorder, and the concomitant use of prescribed analgesia medications are common issues that adversely impact this population. This complex pharmacological profile puts them at added risk of intentional and unintentional injuries [23–25], which further increases the risk of MFD and chronic pain. Unregulated drug use is one option utilized to self-manage pain and suffering [26, 27], but the current contaminated supply of toxic drugs puts those who use drugs at risk of accidental drug poisoning, overdose, and death [28, 29]. In British Columbia, toxic drug poisoning overdoses are currently at an all-time high, with 2,511 deaths in 2023 and 2,253 deaths in 2024 [30, 31]. Poor access and availability of care as well as reduced trust in health care establishments and clinicians often leave people living with social and health inequalities with inadequate pain care. To reduce the need for unregulated drug self-management, this population requires a holistic, trauma-informed, patient-centered, interdisciplinary approach that prioritizes nonpharmacological, nonopioid strategies to achieve some comfort from chronic pain and suffering [32].

The aim of this study was to evaluate the impact of myoActivation care as a component of chronic pain management in the DTES population by investigating changes in pain intensity, quality of life, physical function, need for prescribed analgesic medications, and self-reported unregulated drug use. While there is some evidence for the benefit of myoActivation in pediatric pain care [5, 13, 33, 34], its effectiveness has not been previously studied in this population. The study included quantitative and qualitative methods. This manuscript will report on the quantitative findings; the qualitative findings will be reported separately [35]. The primary quantitative aim was to examine if the inclusion of myoActivation as part of routine clinical care led to a significant improvement in the Pain, Enjoyment of Life, and General Activity (PEG) score; specifically, a 30% reduction in the PEG score over the 24-week course of the study was considered to represent a clinically important benefit [36, 37]. Secondary outcomes included the Pain Catastrophizing Scale (PCS), the Pain Self-Efficacy Questionnaire (PSEQ), and self-reported drug and analgesic use over the 24-week period. This study does not examine the individual components of myoActivation.

## 2. Materials and Methods

### 2.1. Study Design and Approval

This quantitative study was a nonrandomized, prospective, longitudinal, observational, clinical study. Baseline outcome measures were followed up by repeat observations at 4, 12, and 24 weeks from the initial assessment. The study was approved by the University of British Columbia Clinical Research Ethics Board (H19-02567, PI Barb Eddy, approved January 9, 2020). This manuscript has been prepared in accordance with the strengthening the Reporting of Observational studies in Epidemiology (STROBE) guidelines [38].

### 2.2. Setting

The Vancouver Community Pain Service (VCPS) is located in the DTES and utilizes a person-focused, trauma-informed approach to address the unique health care challenges of a population living with social and health inequities. The VCPS provides chronic pain care to all Vancouver community primary care patients, offering interdisciplinary care including myoActivation, counseling, and physiotherapy services. Patients accessing the VCPS choose their initial intake service and can then opt to access other services according to their preference.

### 2.3. Enrollment and Consent of Study Participants

Patients were eligible for enrollment in the study if they were registered with one of six Vancouver Community Primary Care clinics, had chronic pain (pain lasting > 3 months), and had a myoActivation intake appointment scheduled at the VCPS. Patients were excluded if they were non-English-speaking or had received any one of the following interventions for pain management in the preceding 3 months: dry needling, trigger point injection, physiotherapy, massage, joint injection, or chiropractic therapy. Participants utilizing analgesia medications or opioid replacement therapy or who reported substance or alcohol use were not excluded.

Consent for myoActivation treatment at the VCPS is covered by the patients' primary care general consent. Patients presenting to the pain service for myoActivation were approached by a research assistant prior to seeing the treating myoActivation clinician and invited to be part of the overall study. Study consent was obtained in writing just for collection of baseline data, follow-up data, and follow-up interviews.

Once enrolled, baseline data collection began, and the participants were informed of their weekly follow-up timeline. The myoActivation clinician was unaware which patients were enrolled in this study. An honorarium in the form of a gift card of $30 was offered to participants at each data collection follow-up meeting. After enrollment, participants could access other clinic services, including counseling and physiotherapy.

### 2.4. Clinical Procedures: myoActivation® Assessment and Treatment

Participants in the study underwent myoActivation assessment and needling according to routine VCPS clinical practice. Whether enrolled in the study or not, the electronic medical record (EMR) of all patients were reviewed to assess for comorbid conditions, mental health diagnoses, substance use, medications, and imaging. All patients underwent an assessment that included medical history, injury history, and examination. Pain neurobiology, MFD, and myoActivation care were discussed with patients within the context of the biopsychosocial model of pain care [39].

**Figure 1:**
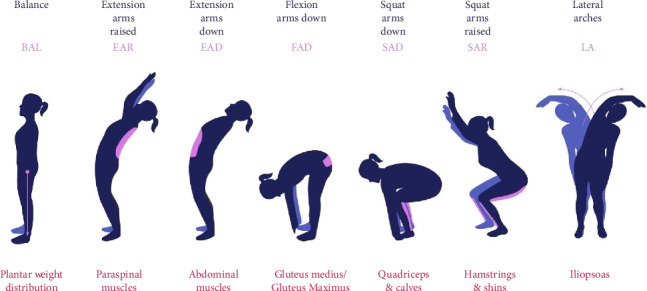
myoActivation assessment is based on seven core BASE (Biomechanical Assessment and Symmetry Evaluation) movement tests.

myoActivation assessment begins with an injury history (the Timeline of Lifetime Trauma [TiLT]), which details the collective history, as far back in time as the patient can remember, of past motor vehicle accidents, fractures, sprains, falls, tailbone injuries, burns, bites, all surgical procedures, and other scars (e.g., chicken pox or acne). The TiLT informs the clinician of the location of scars and soft tissue injuries that may contribute to MFD [6]. The myoActivation clinician then assesses posture and observes the patient performing a standardized set of Biomechanical Assessment and Symmetry Evaluation (BASE) movement tests (Figure 1). The most restricted or painful BASE test directs the myoActivation clinician to the most likely soft tissues impacting MFD. The myoActivation clinician amalgamates the information from the TiLT, posture, BASE tests, observation, and palpation to determine the soft tissues that will be treated first. These tissues are treated with a needling technique using a fine-gauge hollow-bore cutting-tip needle. The needle is inserted and immediately withdrawn for treatment of active muscular trigger points and palpable pain points in fascia. The needling technique for scars has been previously described [6]. No medication or solution is injected. The BASE tests and palpation are then repeated after each treatment to determine the most dominant MFD area that needs to be treated next. The number of needle insertions in each myoActivation session depends on individual treatment needs, tolerance, and response.

myoActivation is a longitudinal process; serial weekly sessions are necessary to unravel and resolve multiple sources of MFD. The required number of serial myoActivation sessions is variable and again based on individual need. Multiple sources of MFD are treated until chronic pain and restriction have resolved or minimized to a new stable state associated with improvements in physical functioning and quality of life. Risks of all needling techniques include discomfort at the site of needling, bruising, dizziness, light-headedness, postcare fatigue, muscle aches, emotional responses, and very rarely, pneumothorax when needling close to the lung fields. myoActivation clinicians have observed, and their patients have reported, that treatment frequently results in immediate changes in pain, flexibility, and ease of movement [35]; preliminary work has been undertaken to establish the methods required to verify these reports [5].

Participants were offered weekly myoActivation sessions following the initial intake visit. While all participants were eligible to receive free services from other interdisciplinary VCPS team members, not all participants wanted to engage in other care initially. Participants may hesitate to enter into talk therapy or physiotherapy for reasons related to past experiences or trust. Mobility restriction can deter a person from movement therapy, and body tension, somatic symptomatology of PTSD, and poor mind/body connection may create barriers to concentrating on cognitive and emotionally related therapy. As myoActivation treatment continued, it was observed clinically that mobility frequently improved and somatic PTSD symptoms lessened, and some participants were able to gain trust in themselves and then had the possibility of engaging in other mind/body work. This new sense of self offered opportunity for referrals to other interdisciplinary team members when the patient was ready and if clinically relevant.

Participants were discharged from myoActivation treatment when they had achieved their goals related to function, pain intensity, quality of life, or when the myoActivation clinician determined that MFD was no longer a significant factor in the patient's presentation of pain or suffering. This decision was based on the participant's response to needling, including anticipated changes to the BASE tests and the patient's report of improvement in pain and restriction.

### 2.5. Data Collection and Outcomes

Baseline data were collected at Week 1, after consent to the study but prior to the participants' initial assessment with the myoActivation clinician. Follow-up data were collected at 4 weeks, 12 weeks, and 24 weeks from the participants' first myoActivation session, including the number of treatments they received between each follow-up (even if none). Follow-up was conducted by telephone or in-person at the clinic before their myoActivation session if the participant had a scheduled appointment on the same day.

Some data were collected from the EMR, including the number of myoActivation and physiotherapy treatment sessions. Data on other clinic and external pain services accessed were obtained directly from the participants by self-report. Also, if the research assistant was unable to contact a participant at a scheduled follow-up time point, other outcome data were collected from the EMR where available; these included PEG score, substance use, and pain medication prescriptions. Participants' TiLT and BASE test findings were not recorded in the study dataset.

#### 2.5.1. Primary Outcome

PEG is a self-report measure of (1) Pain intensity; (2) interference with Enjoyment of life; and (3) interference with General activity. Validity and responsiveness data have been reported for this measure [36, 40], and it has been widely adopted in primary care clinics [41]. Each dimension is scored on a 0–10 scale. The total score is the average (mean) of the 3 values, ranging from 0 to 10, with a lower score indicating a better pain outcome or experience. The PEG was deemed an appropriate tool for the DTES population given the tool's simplicity and minimal time required for completion; it was already routinely administered by some VCPS clinicians. A 30% decrease in PEG total score was considered a clinically meaningful change [42].

#### 2.5.2. Secondary Outcomes

The PCS is a self-report scale that measures a person's cognitive-affective response about the threat of their pain, including their sense of helplessness, rumination, and magnification of pain. Each of the 13 dimensions is scored 0–4 (0 = *not at all*, 4 = *all the time*) [43, 44]. The total score is the sum of these dimensions: minimum 0, maximum 52; a lower score indicates less pain catastrophizing tendencies.

The PSEQ is a validated self-report scale designed to assess the confidence of performing the activities of daily life in people with a lived experience of chronic pain. Validity and responsiveness data have been reported for this measure [45, 46]. Each of the 4 dimensions is scored 0–6 (0 = *not at all confident*, 6 = *completely confident*). The total score is the sum of these dimensions: minimum 0, maximum 24; lower scores indicate less self-efficacy beliefs.

Demographic data collected from participants and the EMR included age, gender, and medical history, including concurrent opioid use disorder ± opioid replacement treatment, substance use disorders, and mental health disorders (anxiety, depression, PTSD, and/or psychotic disorders). In addition, we collected the following baseline data from participants and the EMR: number and location of pain sites; housing; financial status (ministry assistance, employed, other); over-the-counter and prescribed analgesic medications as well as dispensing plan (daily witnessed, daily dispensed, weekly dispensed, other). Concurrent pain services utilized by the patient after consent to this study were recorded. All data were de-identified and stored in a Research Education Data Capture (REDCap) database [47].

Data on participants' substance use (alcohol, cocaine, crack, cannabis [marijuana], crystal methamphetamine, and opioids) and prescribed analgesic medications (acetaminophen, gabapentin, nonsteroidal anti-inflammatory drugs, and opioids) were collected at each study time point from the EMR and from self-reported change in substance and analgesic use (less, same, or more).

### 2.6. Statistical Analysis

Our sample size target was based on (a) feasibility during the study time frame; (b) being consistent with studies of other nonpharmacological interventions [48–50]; and (c) a sample size calculation based on existing PEG data. That is, in the development and validation of the PEG scale in 500 primary care patients with chronic musculoskeletal pain, Krebs et al. reported a mean (standard deviation) PEG of 6.1 (2.2); based on this distribution, a study would require two independent groups of 23 participants each to demonstrate an improvement of a 30% decrease in PEG score, with an alpha of 0.05 and power of 80% [36].

Our original study design aimed to compare three groups of patients undergoing myoActivation and/or physiotherapy at the VCPS: myoActivation-only versus physiotherapy-only versus myoActivation-and-physiotherapy combined based on this sample size calculation. This study design became untenable due to the COVID-19 pandemic and organizational changes at the VCPS. We retained the approved sample of 40 participants for myoActivation enrollment and instead transitioned to longitudinal assessment of participants undergoing myoActivation. We revised our ethics approval and have updated our clinical trials registration (NCT04261959).

Data are presented as median (interquartile range [IQR]) for continuous measures and count (%) for categorical variables. A linear-mixed effects regression model was used to calculate effect sizes (mean differences) in PEG scores between baseline (Week 1) and Weeks 4, 12, and 24; similar analyses were conducted for PCS and PSEQ. This method accounts for the correlation of repeated data points on the same participant and the variability in the number of data points for each participant.

The PCS is a long questionnaire that requires responses to all 13 questions in order to obtain a total score. At baseline (Week 1), some patients were unable to complete all questions, possibly related to attention capacity or trauma triggers. In the case of missing baseline scores for PCS, we substituted the missing baseline total score with the participants' next available complete set of responses (if available) using a next observation carried backward method; we also conducted these analyses without substitution, using a complete case analysis approach.

Statistical analyses were conducted in R Version 4.3.2 (R Foundation for Statistical Computing, Vienna, Austria).

## 3. Results

### 3.1. Study Population

**Figure 2:**
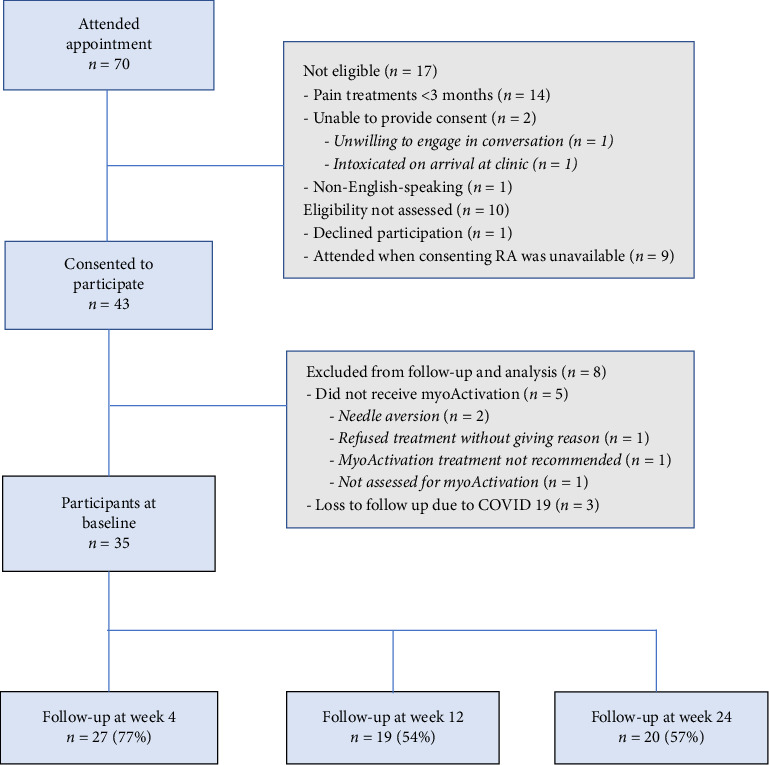
Study flow diagram.

Between February 2020 and March 2022, a total of 70 patients attended a scheduled intake appointment at the VCPS either through primary care or self-referral; 26/70 (37%) who attended were ineligible (Figure 2). A total of 43 participants were enrolled in this study. Three participants attended the clinic during February to September 2020 of the COVID-19 pandemic when study data collection was paused due to clinic closure; these participants were excluded from the analysis. Five participants who consented to participate in the study did not receive any myoActivation treatment (Figure 2). Data collection was completed for the remaining 35 participants. The final follow-up of the last participant took place in August 2022.

The median (IQR) age of participants was 56 years (48–61). At baseline, 18/35 (51%) were homeless, in supported housing, or in a single-room occupancy dwelling; 17/35 (49%) had self-reported or diagnosed PTSD; 24/35 (69%) had some form of substance use disorder; and 31/35 (91%) reported a history of physical or sexual assault (Table 1). All participants reported one or more pain locations: 21/35 (60%) had knee pain; 19/35 (54%) had back pain; 15/35 (43%) had shoulder pain; 14/35 (40%) had hip pain; and 12/35 (34%) reported neck pain. Overall, 29/35 (83%) reported multisite pain. At baseline, many participants reported taking one or more analgesic medications (acetaminophen *n* = 13/35, 37%; nonsteroidal anti-inflammatory drugs *n* = 12/35, 34%; opioids *n* = 12/35, 34%; or gabapentin *n* = 7/35, 20%). Participants also reported taking other substances for either analgesic or nonanalgesic purposes (marijuana *n* = 16/33, 48%; alcohol *n* = 8/33, 24%; opioids *n* = 6/33, 18%; crystal methamphetamine *n* = 2/33, 6%; or cocaine *n* = 1/33, 3%; 2 participants preferred not to answer).

### 3.2. myoActivation Treatments

The median (IQR) number of myoActivation treatments was 4 (2.5–8.5) [range 1–17]. Scars were treated in 26/35 (74%) of participants. A somatic reaction to needling of the scars was experienced by 24/26 (92%), including dizziness, light-headedness, nausea, and pain. In addition, 14/26 (54%) had an emotional response to having their scars treated (crying, recall of traumatic memories/events). One participant experienced dissociation (disconnection between a person and their thoughts, feelings, memories, or identity). Another declined to have their scars treated due to anxiety and worries about potential reactions. No somatic or emotional reactions were noted for the 9/35 (26%) patients who did not have scars treated, and no other adverse events were reported during the study.

### 3.3. Clinic Services Accessed

All study participants chose myoActivation as their preferred initial VCPS service. After starting with myoActivation, some participants also accessed the physiotherapy service after study enrollment: 4/35 (11%) participants by the 4-week follow-up; 7/35 (20%) by 12 weeks; and 10/35 (29%) by 24 weeks. Some participants also self-reported accessing the counseling service after study enrollment: 4/27 (15%) participants by the 4-week follow-up, 6/19 (32%) by 12 weeks, and 7/20 (35%) by 24 weeks.

### 3.4. Primary Outcome: PEG

**Figure 3:**
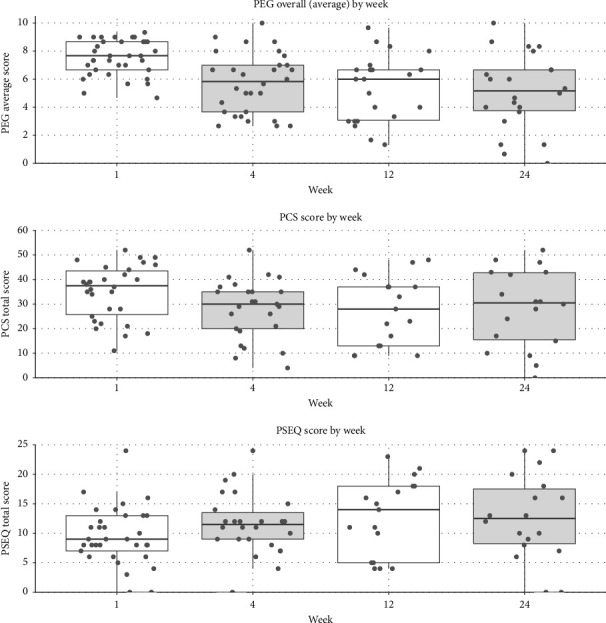
Study outcomes at baseline (Week 1) and follow-up (Weeks 4, 12, and 24), including PEG (Pain, Enjoyment of Life, and General Activity, top subplot) scores, PCS (Pain Catastrophizing Scale, middle subplot), and PSEQ (Pain Self-Efficacy Questionnaire, bottom subplot); data are displayed as box plots overlaid with dots showing individual scores.

At baseline, all participants provided a PEG score, with a median (IQR) score of 7.7 (6.7–8.7). PEG scores improved at each follow-up, with a mean difference from baseline of −1.8 (95% CI −2.6 to −0.9, *p* < 0.001) at Week 4, −2.1 (95% CI −3.1 to −1.1, *p* < 0.001) at Week 12, and −2.5 (95% CI −3.5 to −1.5, *p* < 0.001) at Week 24, a clinically significant [32%] improvement over the study period (Figure 3).

### 3.5. Secondary Outcomes: PCS, PSEQ, and Drug Use

At baseline, four participants were unable to provide complete answers to the 13-item PCS questionnaire, rendering their total PCS scores unavailable; we substituted the missing baseline total PCS scores of three of these four participants with their next available complete set of responses, but one participant did not have a PCS score available at any follow-up point, and their observations were excluded from the analysis of PCS data. The median (IQR) PCS score was 36.5 (26.5–43.5) at baseline. PCS scores improved at each follow-up, with a mean difference from baseline of −5.4 (95% CI −9.5 to −1.4, *p*=0.009) at Week 4, −5.9 (95% CI −10.6 to −1.3, *p*=0.013) at Week 12, and −5.6 (95% CI −10.2 to −1.1, *p*=0.016) at Week 24, corresponding to a 16% improvement over the study period (Figure 3). Complete case analysis yielded similar results.

At baseline, all participants provided a PSEQ score, with a median (IQR) score of 9 (7–13). PSEQ scores generally improved at each follow-up with a mean difference from baseline of 2.2 (95% CI −0.2 to 4.5, *p*=0.076) at Week 4, 3.3 (95% CI 0.5 to 6.0, *p*=0.022) at Week 12, and 3.5 (0.8–6.2, *p*=0.013) at Week 24, corresponding to a 36% improvement over the study period (Figure 3).

At the Week 24 follow-up, 20/35 (57%) participants provided follow-up data on their continued drug use; drug use data for an additional seven (20%) participants were obtained from the EMR. Of these, 9/27 (33%) participants self-reported using less unregulated substance use, 10/27 (37%) self-reported using less unregulated substance use for the purpose of pain management, and 8/27 (30%) self-reported using less analgesic medication.

## 4. Discussion

In this study, myoActivation treatment was a major component of the interdisciplinary management of chronic pain in a population living with social and health inequities. Participants had a median of four myoActivation sessions, and we observed a clinically meaningful 32% decrease in the PEG score, an improvement well beyond the recently reported minimally important difference when applying this measure [51]. This was sustained at the 24-week assessment, indicating a lasting improvement of pain intensity and quality of life and is a major strength of our study. We also demonstrated a 16%–17% reduction in the PCS and a 35%–36% improvement in the PSEQ at the 12- and 24-week follow-up, as well as a self-reported decrease in substance and analgesic use.

These results represent a crucial finding for this population, where there are significant barriers to access and availability of chronic pain care services [14, 16, 17]. myoActivation has the potential to reduce the need to self-medicate with unregulated drugs and therefore reduce the risk of accidental toxic drug poisoning, overdose, and death [28, 29]. Even in this population living with social and health inequities, we demonstrated positive change. Hence, we may have a potential solution to the task set in current Canadian guidelines for a nonpharmacological treatment that improves access to care for people living with chronic pain [15, 32]. These outcomes may be generalizable to all patients with a lived experience of chronic pain.

The relation between pain catastrophizing and pain perception is a two-way process; the perceived threat of pain contributes to adverse pain outcomes, but the experience of pain may very well also promote catastrophic thinking [52, 53]. The PCS was initially designed to assess the multidimensional nature of catastrophic thinking that includes helplessness, rumination, and magnification. High scores on the PCS magnification subscale have been wrongly interpreted as an intensification or overestimation of the pain experience and not the intended PCS use of “tendency to exaggerate the threat value of the pain experience” [44]. Since its development in 1995 [43], the PCS has been used more as a screening tool predicting increased risk of poor pain prognosis and outcomes [44, 54, 55] and less as a tracking tool. We elected to follow the change in the PCS between baseline and follow-ups and found a 16% reduction, though this is less than the 38%–44% decrease, which has been reported to be clinically meaningful for the PCS [56]. The positive response we observed possibly correlates to perceptions of threat and hypervigilance as a symptom of PTSD that was so prevalent in our participants. The novel model of pain care offered by myoActivation may well have instilled a sense of hope for resolution in patients with long-term chronic pain, which contributed to the reduction in their perception of pain as a threat [35]. Consequently, we caution against the use of the PCS solely as a screening tool to avoid biased judgment of a patient's pain experience and potential treatment outcomes.

A higher PSEQ score reflects stronger beliefs of self-efficacy in a patient's ability to complete day-to-day tasks such as socializing, work, and household chores. There is limited empirical evidence to justify what constitutes a clinically meaningful change in the PSEQ [57, 58]. We observed a 35%–36% improvement at the 12- and 24-week timepoints, although the wide confidence intervals imply a significant variation between participants. The improvement at the later timepoints may reflect the sequential changes that occur in myofascial tissues and function with ongoing myoActivation sessions, and, therefore, confidence in living with less pain was realized later. In addition, this population may require time to trial and gain trust in their embodied changes.

Many people with concurrent chronic pain, mental health, and substance use disorders resort to unregulated substances to self-manage their pain and suffering. Eight years of provincial administrative data investigating DTES substance use disorders reveals a prevalence of 55% for opioid use disorder [59]. We found that over two-thirds of our participants had some form of clinically diagnosed substance use disorder, and over half had two or more. Approximately one-third of our participants self-reported using less unregulated substances, less unregulated substances for the purpose of pain management, or less pain medication over the course of the study. The small numbers and self-reported nature of this outcome suggest this should be interpreted with caution. However, this preliminary evidence suggests that myoActivation contributes to improved pain and reduced suffering, helping to support reduced analgesic and unregulated drug use. This is particularly meaningful in this population and warrants further study.

DTES residents are at higher risk of experiencing or having experienced violence, sexual assault, and emotional trauma [60, 61]. A review of our participants' TiLT identified an extremely high prevalence of physical assault, sexual assault, PTSD, and other mental health concerns (Table 1). Incorporating myoActivation into care requires careful consideration of these issues. The VCPS adopts a person-focused, trauma-informed, and compassionate approach to gain trust and engagement, but aspects of care may need to be tailored to the unique challenges of the DTES population. For example, a full injury history might not be appropriate on a first visit to minimize reliving traumatic experiences. Similarly, there may be a reluctance to remove clothing and feelings of vulnerability when moving and changing posture for myoActivation movement tests and treatments. myoActivation therapy may trigger the recall of traumatic memories and induce somatic or emotional responses [35]. To mitigate the risk of distress or self-harm during and following treatment, it is important to understand and support the person's capacity to emotionally regulate and to assess engagement in professional mental health care and support from friends and other community resources. Additionally, changes in bodily sensations, pain intensity, or pain location posttreatment must be explained so that they are not interpreted as harm, opioid withdrawal symptoms, or a new chronic pain experience.

The majority of our participants had needling of scars, and over half of those who had their scars treated exhibited somatic or emotional reactions. Two of the five participants who provided consent declined to proceed with myoActivation due to worries of triggering a memory of past psychological or physical injury, such as abuse. Emotions and memory of traumatic events have been increasingly linked to the fascial system [62, 63], with bidirectional communication between the peripheral and central nervous systems [64]. After an emotional response to needling of soft tissues, positive outcomes of hope, renewed energy, and even improved mood are witnessed and are likely partly responsible for the positive outcomes we observed [35].

### 4.1. Limitations

Our study is subject to several limitations. The study had a small sample size at a single clinic location and followed a nonrandomized, longitudinal observational clinical design with no control group. The sources of bias include variable attendance that is characteristic of the study population [65, 66] and the subsequent missing data due to difficulty with follow-up that may have been further exacerbated by the ongoing impacts of COVID-19. While we experienced significant loss to follow-up, we were able to report drug use data from 77% of our participants at Week 24, almost 6 months after their enrollment, which is a significantly positive outcome in this patient population. Attendance in studies for people living with social and health inequities is impeded because they experience unique everyday life concerns and health issues that may be hectic and unpredictable, disrupting clinical and research participation [67]. Our study sample may have been impacted by selection bias: The patients who attended the initial intake appointment and provided consent to participate may have had more stable housing and access to reliable means of communication and transportation, been more engaged in care, or been less vulnerable to the impact of existing physical and mental health problems compared to those who did not follow up. The VCPS location where the study was conducted was convenient for patients who already lived in the DTES but less so for those who resided outside of this area.

We used the PEG 3-item questionnaire to support patients' capacity to complete the questions. This choice meant we did not have data related to sleep and mood collected on longer pain questionnaires such as the Brief Pain Inventory [68]. We included the PCS and PSEQ questionnaires, as these had already been adopted by the clinic for longitudinal patient assessment. We chose to not add measurements of anxiety or depression, commonly monitored in pain studies, as additional screening tools may have been impractical for this patient population, possibly evident in the limited completion of the PCS questionnaires.

We cannot conclude that all observed improvements were due to myoActivation alone, as the other interdisciplinary care services offered by the VCPS would likely contribute to these improvements. We did not track how many appointments were attended with other care providers after intake, except for physiotherapy and counseling. However, there was limited uptake of physiotherapy and counseling services among our participants during the study. Ideally, future work should consider comparing outcomes for myoActivation patients who are also participating in physiotherapy versus myoActivation patients who are not having physiotherapy—similarly counseling or other interdisciplinary therapies.

A challenge of analyzing data collected from an uncontrolled intervention and a real-time clinical service is that the number of data points will vary according to the frequency of appointments required to meet each individual participant's treatment needs. This was mitigated using a linear mixed-effects model in our analysis to account for variability between and within participants' data. We cannot rule out the possibility that for our study participants, many of whom had a history of physical or sexual assault, simply having a positive engagement with a healthcare provider may have contributed a beneficial effect on their pain outcomes.

This study did not specifically evaluate the individual myoActivation components (BASE tests, TiLT, postural assessment, palpation, and patient self-report of painful and restricted movement) or their relative contribution to the overall clinical assessment and decision-making. Preliminary work has been conducted to identify objective measures for range of motion in patients performing BASE tests [5], but further work is required to investigate the validity or reliability of these individual assessment techniques.

## 5. Conclusion

This study provides preliminary evidence that myoActivation, a novel nonpharmacological tool, helped to relieve chronic pain in a population living with social and health inequalities in Vancouver's DTES, many of whom had suffered from previous emotional and physical assaults. myoActivation is a low-cost technique that can be easily applied as a point-of-care pain treatment by a primary care clinician who has a longitudinal relationship with the patient and capacity to wean medications when able. Our findings should kindle a great deal of curiosity as to the impact of MFD (scars, muscles in sustained contraction, skin in tension, and fascia in tension) in all chronic pain presentations, not just our DTES participants. This pilot study provides valuable preliminary data to inform a future prospective multicenter longitudinal study to further assess the impact of myoActivation in populations living with social and health inequalities. Given the high prevalence of chronic pain in the general population and limited options for drug-free treatment, these findings are encouraging and indicative of a potential solution to improve the lives of many people with a lived experience of chronic pain.

## Figures and Tables

**Table 1:** Participant demographics.

Characteristic	For cohort (*N* = 35) *n* (%) or median (IQR)	
Age (years)	56 (48–61)	
Gender		
Male	19 (54%)	
Female	14 (40%)	
Transgender or nonbinary	2 (6%)	
Income		
Disability	26 (74%)	
Welfare	3 (9%)	
CPP/OAS	5 (14%)	
Other source of income	8 (23%)	
Housing at baseline		
Homeless	2 (6%)	
SRO dwelling	4 (11%)	
Supported housing	12 (34%)	
Other housing	17 (49%)	
Physical trauma		
Physical assault (excluding sexual)	22 (63%)	
Sexual assault	10 (29%)	
Mental health	Diagnosed	Received treatment
Anxiety	14 (40%)	11 (31%)
Axis 1	6 (17%)	4 (11%)
Depression	20 (57%)	16 (46%)
PTSD	17 (49%)	7 (20%)
Substance use disorders	At baseline	In the past
Alcohol use disorder	8 (23%)	9 (26%)
Cannabis dependence	6 (17%)	2 (6%)
Opioid use disorder	14 (40%)	2 (6%)
Stimulant use disorder	7 (20%)	6 (17%)
Substance use disorder (any)	24 (69%)	5 (14%)

Abbreviations: CPP/OAS = Canadian pension plan/old age security, PTSD = posttraumatic stress disorder, SRO = single-room occupancy.

## Data Availability

The data that support the findings of this study are available from the corresponding author upon reasonable request. Study data are available on request from the corresponding author.

## Supporting Information

The myoActivation Process—This appendix provides a description of the myoActivation process, including details of the assessment and therapeutic technique.
