# Supplementary material for: The Impact of a Novel Methodological Process for Needling Scars, Fascia, and Muscles in the Management of Myofascial Dysfunction and Chronic Pain in a Population Living With Social and Health Inequities: Quantitative Findings From a Longitudinal Observational Pilot Study
Source: Pain Res Manag. 2025 Sep 28;2025:8567447. doi: 10.1155/prm/8567447 (PMC12497526; doi:10.1155/prm/8567447)
Supplement: Supporting Information — Additional supporting information can be found online in the Supporting Information section. [file 8567447.f1.pdf]

The myoActivation Process

Table of Contents

myoActivation® summary ***2***

Assessment ***3***

Timeline of Lifetime Trauma (TiLT) **3**

Observation **4**

Postural Assessment **4**

Core Biomechanical Assessment and Symmetry Evaluation (BASE) tests **4**

Identification of the area of greatest myofascial dysfunction **5**

Inspection **7**

Palpation **7**

Therapeutic technique ***7***

Needling technique **7**

Release of scars **8**

Activation of muscles in sustained contraction **8**

Reduction of fascial tension **8**

Trauma informed care **8**

Catenated cycles **9**

Deciding factors on when to stop in any one myoActivation session **10**

Aftercare instructions ***10***

Number of sessions needed to unravel multiple sources of myofascial dysfunction ***11***

Clinically observed benefits of myoActivation ***11***

Acknowledgement ***12***

Further Reading ***12***

myoActivation® summary

Myofascial dysfunction occurs in response to past injuries or surgeries, where the soft tissue injuries from these past traumas results in scars, skin tethering, muscles in sustained contraction or densified and tight fascial tissues. These soft tissue impacts can occur either in isolation or as a combination of all these factors. Myofascial dysfunction is commonly overlooked as a contributing etiological factor in chronic pain presentations. myoActivation® is a unique structured system of assessment and treatment designed to reduce myofascial components of chronic pain. Due to the myofascial connections and biotensegral properties of the human body a core principle of myoActivation is that the site of pain is often not the source of pain.

myoActivation assessment starts with a detailed injury history dating as far back in time as can be remembered. This Timeline of Lifetime Trauma (TiLT) includes the collective history of past motor vehicle accidents, fractures, sprains, falls, tailbone injuries, burns, bites, major and minor surgical procedures and, other scars. The myoActivation practitioner then assesses posture and observes standardized functional movements called Biomechanical Assessment and Symmetry Evaluation (BASE) tests. The most restricted or painful BASE test directs the myoActivation practitioner to the most likely tissues impacting myofascial dysfunction. After inspecting the identified area for skin tethering or scars the clinician then examines the area for palpable pain points to identify dominant areas of myofascial tension. The practitioner amalgamates the information from the reported TiLT, posture assessment, BASE test assessment and examination to direct care.

myoActivation intervention is a needling technique utilizing fine gauge hollow-bore, cutting-tip hypodermic needles with no injectate. Needling at the palpable pain points is designed to release myofascial tension. The BASE tests and palpation are repeated after each treatment (catenated cycles) to determine the next most dominant myofascial site that needs to be treated next.

The number of myoActivation sessions required to loosen sources of myofascial dysfunction vary depending on each individual. myoActivation sessions are usually spaced 1-2 week(s) apart. Usually, 4-8 myoActivation sessions are required to unravel multiple sources of myofascial dysfunction in a longitudinal process, but more sessions may be required if there is an ongoing trigger for myofascial dysfunction.

> **Example**: a patient with a history of previous significant burns and scarring will likely need many more sessions for ongoing release of soft tissues.

The process focuses on improvements in the core BASE tests and then improving regional BASE tests for specific areas such as the neck, shoulder or jaw. After treatment immediate aftercare includes regular posture change to allow the nervous system to adjust to new neural circuitry and the treated tissues to recover. Long term aftercare requires attention to increasing physical activity and strengthening soft tissues.

Risks of myoActivation include discomfort at the site of needling, bruising, dizziness, light-headedness, emotional responses, post care fatigue, muscle aches, changing sensation of pain and, very rarely pneumothorax when needling over the chest wall. myoActivation clinicians have observed and patients report that treatment results in immediate improvement or changes in pain, flexibility, and ease of movement, often this is appreciated after the first session but with multiple sources of myofascial dysfunction this may not be realised until session two or three in this longitudinal process.

Assessment

Timeline of Lifetime Trauma (TiLT)

The TiLT is essentially a detailed history from as far back as the patient can remember of any motor vehicle accidents, fractures, sprains, falls, tailbone injuries, major surgeries, minor surgeries, burns, bites, or other scars. The mechanism of each injury is sought to help elucidate which soft tissues may have been impacted. A high-level understanding of the physical, emotional and social impacts of each incident is heard to appreciate the potential for trauma related somatic symptoms. The associated healing process of any scar is essential to determine their significance in the pain presentation. Clinical experience suggests that infection during a healing process and injuries sustained at the youngest age appear to have significant impact on myofascial dysfunction. An important enquiry in the myoActivation history is to ask the patient what they consider to be their greatest physical trauma. Scars are particularly relevant even if they appear to look normal or are very small.

These details help the myoActivation clinician determine what soft tissue may have been injured and therefore where the patient may have myofascial dysfunction.

> **Example**: a scar in an area hidden by clothing may have never been found if the myoActivation clinician did not ask and know these details.

The TiLT is integrated with the subsequent examination findings to help determine their significance in the current chronic pain presentation.

Observation

The myoActivation clinician observes the patient for asymmetry in gait, seated stance or standing posture during the clinical encounter.

> **Examples**: a knee in flexion during the swing phase of walking may indicate that the ipsilateral hamstrings or lateral abdominal wall muscles are in sustained contraction, a seated stance where the patient sits with the feet internally rotated may indicate tightness in the medial fascial plane or the adductors are in sustained contraction, or, when standing pelvic rotation may indicate that the ipsilateral TFL, iliopsoas, and rectus femoris or contralateral gluteus medius are in sustained contraction.

Initially, the patient is asked to identify the location of their perceived pain; this helps direct the examination and is used as an index for subsequent treatment effect. Where the patient identifies or indicates is the perceived site of pain is rarely the tissue that is responsible for the true origin of pain.

> **Example**: lower quadrant abdominal pain may originate from an ipsilateral quadratus lumborum muscle in sustained contraction.

Postural Assessment

The clinician observes the standing postural and notes if there are any differences between the right and left sides of the body reviewing; feet (e.g. pronated, elevated little toe, clawed toes), knees (e.g. hyperextended or hyperflexed), level of the hips, any pelvic rotation or tilt, shoulder height, as well as any shift or tilt of the torso and head position).

> **Examples**: hyperextended knees indicate that the quadriceps muscles are in sustained contraction, a head forward posture would indicate that the sternocleidomastoid, platysma or anterior abdominal wall musculature was in sustained contraction or that the sternal fascia is tight.

Core Biomechanical Assessment and Symmetry Evaluation (BASE) tests

These tests are used to screen a patient's body for the true origin of pain. BASE tests compartmentalize the true origin of pain to a defined anatomical region (Figure 1).

First the patient is asked about the perceived weight distribution on their feet (right = left, back, front or central and outside inside or central). The talus has no muscular attachments so it functions as a ball and socket joint around which the skeleton sways depending on the distribution of myofascial forces. In humans the centre of the body mass is normally located anterior to the S2 vertebrae. In an erect stance if

there is no significant anatomical postural distortion, the center of mass or gravity will be evenly distributed between the feet and over each plantar surface. Therefore, if one foot feels heavier than the other then there is a shift of the centre of mass or gravity towards that side of the body.

Example: if weight is perceived to be more on the right foot, then there is likely contracted musculature in the right leg 'pulling' the pelvis to the right and shifting the centre of mass to the right.

One of the aims of the therapeutic component of myoActivation is to change the perceived balance of weight on the feet to be more in equilibrium.

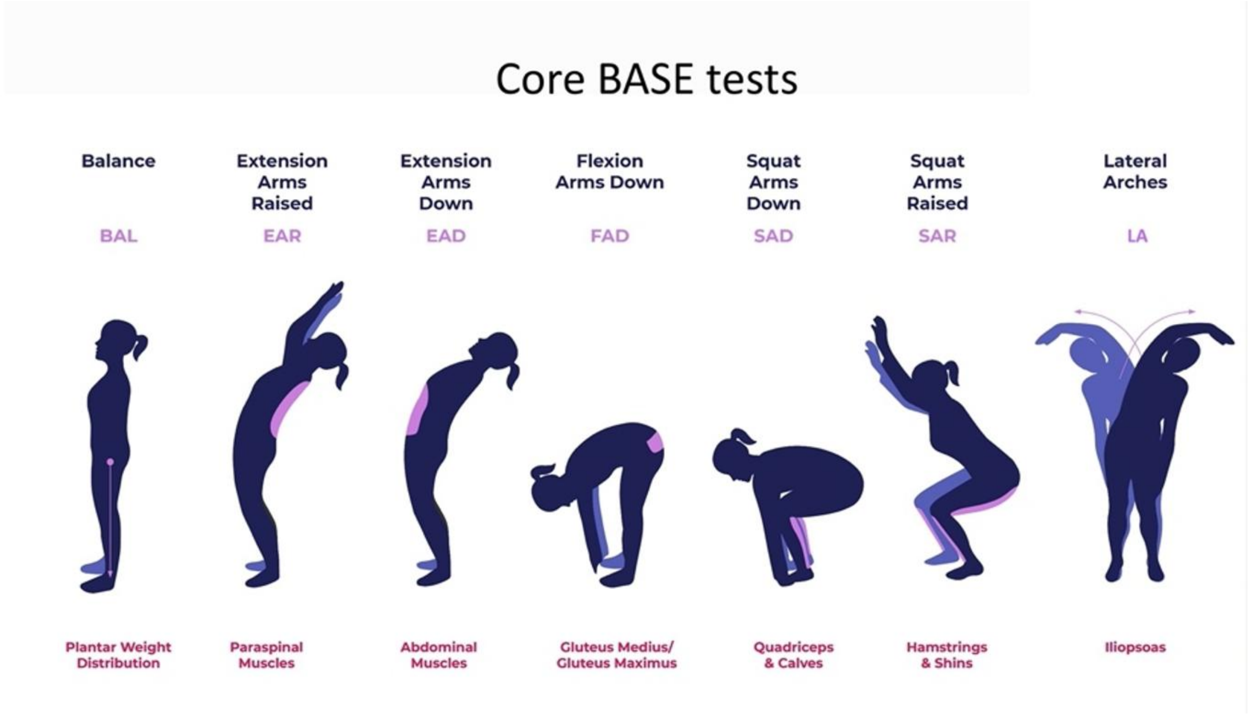

***Figure 1**: Biomechanical Assessment and Symmetry Evaluation (BASE) tests.* *The seven core tests include (left to right): balance (BAL), extension arms raised (EAR), extension arms down (EAD), flexion arms down (FAD), squat arms down (SAD), squat arms raised (SAR), and lateral arches (LA). The related tissues are described below each movement demonstration, in red text.*

Identification of the area of greatest myofascial dysfunction

The subsequent tests are used to identify the most painful or restrictive BASE test. Administering these core BASE tests is a quick assessment. The most painful or restrictive BASE test identifies the tissues that are most likely contributors to the perceived pain. Even though the individual BASE tests are common human movements, the coordinated use of these movement tests to define anatomical areas that are the true origin of pain is unique. Each test defines a specific muscle group or body area as shown in Figure 1.

The most painful or restrictive test generally provides a clear indication of a starting point for treatment when a patient has multiple sites of pain or widespread pain.

Example: if EAR is the most restricted or painful test for an individual patient then the myoActivation clinician will first inspect and examine in the paraspinal region.

The individual BASE tests are explained below:

- **Extension Arms Raised (EAR):** The patient is instructed to bend backwards from the hips with their arms overhead. Wherever pain is perceived by the patient in this posture, the true source of pain originates in the paraspinal muscles.
- **Extension Arms Down (EAD):** The patient is instructed to arch backwards from the hips with their arms down. Wherever pain is perceived by the patient in this posture, the true source of pain originates in the abdominal muscles.
- **Flexion Arms Down (FAD):** The patient is instructed to flex forward with straight knees and bend forwards to wherever they can reach comfortably. The patient is questioned in regards specifically to pain in the low back. If pain is perceived in the low back in this posture, the true origin of pain is in the medial gluteus medius and/or gluteus maximus muscles.
- **Squat Arms Down (SAD):** The patient is instructed to squat with their arms by their side to where they can crouch comfortably. If a patient has a very restricted squat, their technique in performing the squat can be improved by instructing them to drive their buttocks backwards. A deeper squat will invariably result due to increased pelvic rotation from this maneuver. Where ever pain is perceived by the patient in this posture, the true origin of pain is in the quadriceps or calf muscles. If the pain is perceived to be in the upper leg, then the quadriceps will be the pain source. If in the lower leg, then the gastrocnemius and/or soleus will be the source.
- **Squat Arms Raised (SAR):** The patient is instructed to squat with their arms overhead to where they can crouch comfortably. Where ever pain is perceived by the patient in this posture, the true origin of pain is in the hamstrings or tissues overlying the shin. If the pain is perceived to be in the upper leg, then the hamstrings will be the pain source. If the pain report is the lower leg, then the medial tibial fascia or soft tissues will be the source.
- **Lateral Arches:** The patient is instructed to bend to the side (like a ballerina). Wherever pain is perceived by the patient in this posture, the true origin of pain is in one of the iliopsoas muscles.

Inspection

The myoActivation clinician inspects the region of the most painful or restricted BASE test for any asymmetrical features, tethered skin, skin creases, fascial lines of tension or any scars, even tiny ones.

> **Example**: if SAR is the most restricted or painful BASE test with pain reported in the lower legs doing that movement then any scar on the shin will be significant even if it is a tiny chicken pox scar or seemingly insignificant scar from a childhood injury.

Palpation

The technique of palpation requires rolling motion to be used, applied using both thumbs or index fingertips simultaneously, on symmetrical tissues to compare right and left sides. Differences between right and left may be apparent by the patient's physical reaction, patient's verbal report, and/or by sensory feedback to the examiner from digital pressure. That sensory feedback of the clinician improves with experience.

The goal in palpation of soft tissues is to identify increased density, which is painful to the patient and feels different to the clinician when comparing the same tissue on the other side. In most instances, when increased density of a soft tissue is identified, the patient will express or react to the noticeable increase in discomfort or pain associated with palpation of the abnormal tissue. The painful palpable pain points are very important as the indicator of a myofascial source of pain.

Therapeutic technique

Needling technique

Needling is performed with fine gauge (25-30 gauge) hollow-bore cutting-tip needles, that are similar to the needles used for bloodletting. The size of the needle varies depending on the tissue to be treated and the body habitus of the patient. No injectate is used. Care with size of needle is especially important when needling around the chest/neck to prevent a pneumothorax. The technique of needling varies dependant on whether a muscle is to be activated, fascia released, scar released or skin crease/tethering to be released. A detailed knowledge of anatomy and training in myoActivation is essential to safely and effectively needle soft tissues to relieve myofascial dysfunction. The myoActivation clinician also needs to assess needle safety for any patient with a current or past intravenous drug use and associated body scars.

Release of scars

The myoActivation needling technique for release of scars involves the sequential insertion of 30g hollow bore needle in the line of the scar and in any areas of densification around the scar performing multiple perforations approximately 3 mm apart. Patients often report a “biting sensation” with needling which occurs even if the scar has been treated with appropriately timed topical anaesthetic or pre-needling vapocoolant spray.

Activation of muscles in sustained contraction

The myoActivation technique for activation of muscles in sustained contraction involves sequential palpation of the muscle to find the most painful point (the active trigger in the muscle), this is best done by palpating bilateral muscle groups simultaneously to help the patient and the myoActivation clinician determine the most palpable pain point. A 25–30-gauge hollow-bore cutting-tip needle is quickly inserted into that trigger point and immediately removed. If successful this will be associated with a palpable release and lengthening of that muscle, and may be associated with a muscle twitch (the twitch response), but, a twitch response is not mandatory for a successful activation of a muscle in sustained contraction. Repeat palpation at the same site will not be painful if the muscle has been successfully activated. Needling may need to be repeated if there is another palpable pain point in the same muscle group at a different site.

Reduction of fascial tension

The technique of needling to release fascial tension varies dependant on the type of fascial tension that is observed on inspection or on the palpation findings in the region of the most restricted or painful BASE test. If there is a skin crease or skin tethering these lines of tension are released in a similar technique as indicated above for a scar. If there is a palpable painful density in the fascia then this is released with 3-5 quick insertions to resolve that palpable pain and density.

Scars, skin tethering, dense fascia and muscles in sustained contraction do not exist in isolation so when a scar is released it may be possible that other underlying connected myofascial tissues will be released at the same time. Similarly, when a muscle is activated it is likely that the surrounding fascial coverings will also be released.

Trauma informed care

Trauma informed care recognises that past traumatic experiences are closely linked with subsequent health problems. A “trauma-informed approach incorporates three key elements: (1) realizing the prevalence of trauma; (2) recognizing how trauma affects all individuals involved with the program,

organization, or system, including its own workforce; and (3) responding by putting this knowledge into practice" (SAMHSA, 2012). Key components include meeting client needs in a safe, collaborative, and considerate manner; avoiding retraumatize people who are seeking help or receiving services; building on the strengths and resilience of clients in the context of their environments and communities; and endorsing trauma-informed principles throughout the continuum of care. A trauma informed approach requires that the myoActivation clinician recognizes the patient's need for choice and agency in the whole myoActivation process and unravel an individual's pain and suffering in a manner that is most appropriate for them.

Examples: some patients may not want to remove clothing, some may feel vulnerable with certain movements or positions of treatment, the number of needles in any one session needs to be carefully assessed and monitored, allowing patients to refuse certain aspects of care, and permission to touch before any examination.

Establishing the TiLT in a compassionate way is often the first part of this process. myoActivation treatment needs to be applied with a trauma informed approach that supports the patient's emotional well-being. It has been observed by myoActivation clinicians that when traumatically injured soft tissues are needled some patients may recall past events or experience emotional releases. These experiences are very variable but range from mild reflections to vivid recall. These experiences may also evoke memory of associated scents and sounds, emotional awareness and crying. The patient is usually surprised by these responses to needling and may need time to work through the experience. If strong emotional responses are anticipated, myoActivation sessions can be delayed while the patient prepares for the treatment and organizes for psychological supports. People with lived experience of chronic pain often understand how their emotions and injury history relate to their pain. In the process of unravelling the myofascial components of chronic pain with myoActivation needling the emotional experience associated with relief of tension in the body often improves hence myoActivation applied in a trauma informed manner can play a unique role in holistic healing from chronic pain.

Catenated cycles

Catenated cycles are repeated sequences of BASE testing, palpation and needling that occur in each myoActivation session to unravel the multiple sources of myofascial dysfunction. There may be 1-6 catenated cycles in each myoActivation session. Each cycle usually identifies the next new and different most painful or restrictive BASE test resulting in a new region for inspection, palpation and treatment. Catenated cycles demonstrate to the clinician some or all of the following: visible changes in patient

movement; increase in joint range, greater range of motion, increase in speed of movement, increase in ease, smoothness or fluidity of movement and changes in reported pain with each movement.
For the patient, catenated cycles will demonstrate some or all of the following subjective changes in post-treatment movement: reduction in overall perceived pain at rest and/or movement, reduction or a diffusion in the area of pain, shift in pain location, perception of pain only at end range rather than throughout range, or a different pain focus altogether at a different location that only becomes perceptible when the initial painful site has been treated. Another advantage of the catenated cycles is that the patient has to get up and move after each treatment, which distracts from any discomfort resulting from the treatment process.

Deciding factors on when to stop in any one myoActivation session

It is optimal to end sessions at a successful end-point. These might include: resolution of pain, reduction in pain, improved flexibility, increased fluidity of movement, positive postural changes, or change in the weight distribution of the feet to being more grounded (even plantar weight distribution). Otherwise, the decision during treatment to stop further needle insertions is a clinical judgement that is dictated primarily by the patient's ability to tolerate the procedure. Fatigue, feeling overwhelmed and emotional responses are not uncommon especially during the first treatment session. An important principle is not to do too much at each session, especially the first one. When the origins of the targeted MFD are from a traumatic event (e.g., abuse, assault, major injury), the patient's readiness for needling needs to be carefully assessed in a trauma informed approach as outlined above. Sometimes only one needle insertion can be performed or only one site treated rather than doing more than one catenated cycle in one session. Fewer needle insertions per session are recommended for a patient with complex trauma history, heightened arousal, and poor capacity to regulate their emotions.

Aftercare instructions

Instructions following treatment are directed to promote recovery of treated tissues and prevent symptom regression. Patients are advised to move regularly, with frequent changes in posture (every 10-15minutes) whilst awake in the first 24-48 hours after each myoActivation session. They are also advised to avoid myofascial loading, repetitive exertion, and prolonged postures for 5 days. After this time, they can return to or start graduated activity. The post-treatment response will be an individualized experience for each patient. Multiple factors will govern the outcome resulting from treatment including; degree of pre treatment sedentary activity, physical demands in the workplace, nutrition, patient age, genetically determined responsiveness of soft tissues and, psychosocial factors related to chronic pain.

Number of sessions needed to unravel multiple sources of myofascial dysfunction

It is optimal to schedule 2-4 sessions, one or two weeks apart, to minimize the need to do too much at each individual myoActivation session, minimize discomfort following treatment and to help determine responsiveness. After three sessions, the myoActivation clinician can usually determine if there is sufficient positive response to continue. Serial change is observed in positive responders usually experience less pain so that activities of daily living have improved, BASE tests serially improve in terms of the number that can be achieved without discomfort and in their range of motion. There is a wide range in numbers of sessions required in positive responders, but it usually requires 2-8 sessions to achieve improved range of motion in all BASE tests and resolution of chronic pain. This decision to stop myoActivation sessions is based on the participant's response to needling, changes to the BASE tests and the patient's report of improvement in pain and ability to perform activities of daily living.

Clinically observed benefits of myoActivation

After treatment, the patient may observe changes in their pain, such as a reduction in intensity or a shift to a different area. They might also find that the pain no longer arises during movements that previously triggered it. Movements are easier to complete and are more fluid in their execution. Patients may notice that they feel more balanced in the weight distribution between their feet.

Acknowledgement

Dr Greg Siren has trademarked myoActivation® to preserve intellectual property and the unique standardized innovative process within the auspices of a not-for-profit organization, the Anatomic Medicine Foundation (https://www.anatomicmedicine.org/).

Further Reading

1. Lauder G, West N, Siren G. **myoActivation: A Structured Process for Chronic Pain Resolution** [Internet]. From Conventional to Innovative Approaches for Pain Treatment. *IntechOpen*; 2019. Available from: http://dx.doi.org/10.5772/intechopen.84377
2. Luo J, West N, Lauder GR. **myoActivation®, a Structured Assessment and Therapeutic Process for Adolescents with Myofascial Dysfunction and Chronic Low Back Pain: A Case Series**. *Cureus*. 2024;16(8):e68029. http://dx.doi.org/10.7759/cureus.68029
3. Bhatnagar T, Azim FT, Behrouzian M, Davies K, Wickenheiser D, Jahren G, West N, Leveille L, Lauder GR. **Assessing changes in range of motion in adolescent patients undergoing myoActivation® for chronic pain related to myofascial dysfunction: a feasibility study**. *Front Pain Res*. 2023;4:1225088. http://dx.doi.org/10.3389/fpain.2023.1225088
4. Lauder GR, Huang J, West NC. **Unrecognised myofascial components of pediatric complex pain: myoActivation, a structured solution for assessment and management**. *Curr Trends Med*. 2019;1(1): 1-16. https://www.researchgate.net/publication/342865042
5. Lauder G, West N. **Clinical Insights into the Importance of Scars and Scar Release in Paediatric Chronic Myofascial Pain** [Internet]. Pain Management - Practices, Novel Therapies and Bioactives. *IntechOpen*; 2021. Available from: http://dx.doi.org/10.5772/intechopen.93525
6. Lauder GR, Luo J, Nassaazi J. **Early-Life Trauma as a Trigger for Developmental Myofascial Dysfunction: A Case Report**. *Cureus*. 2025;17(7):e87863. http://dx.doi.org/10.7759/cureus.87863
